# Assessment of Hypertension in Hemodialysis Patients with the Concomitant Use of Peridialytic and Interdialytic Ambulatory Blood Pressure Measurements

**DOI:** 10.3390/life15081290

**Published:** 2025-08-14

**Authors:** Kallistheni Leonidou, Ioannis Kontogiorgos, Christodoula Kourtidou, Eleni Georgianou, Vasileios Rafailidis, Stefanos Roumeliotis, Konstantinos Leivaditis, Elias V. Balaskas, Vassilios Liakopoulos, Panagiotis I. Georgianos

**Affiliations:** 1 2nd Department of Nephrology, AHEPA Hospital, School of Medicine, Aristotle University of Thessaloniki, GR54636 Thessaloniki, Greece; kalialeonidou@hotmail.com (K.L.); ikontogiorgos.med@gmail.com (I.K.); christinakourt@hotmail.com (C.K.); elenigeorgi@hotmail.com (E.G.); st_roumeliotis@hotmail.com (S.R.); konleiv@windowslive.com (K.L.); ebalaskas@hotmail.com (E.V.B.); liakopul@otenet.gr (V.L.); 2 Department of Radiology, AHEPA Hospital, School of Medicine, Aristotle University of Thessaloniki, GR54636 Thessaloniki, Greece; billraf@hotmail.com

**Keywords:** ambulatory BP monitoring, epidemiology, hemodialysis, hypertension, phenotypes

## Abstract

**Background:** For patients on hemodialysis, routine blood pressure (BP) measurements taken shortly before or after dialysis provide inaccurate estimates of the BP load during the interdialytic period. In this study, we used peridialytic recordings in combination with interdialytic ambulatory BP monitoring (ABPM) aiming to provide a more precise assessment of hypertension in a sample of 70 stable hemodialysis patients. **Methods:** The evaluation of hypertension in the study cohort was performed using the following approaches: (i) routine predialysis and postdialysis BP measurements taken by the dialysis-unit staff were prospectively recorded over six consecutive dialysis sessions; (ii) ABPM was performed using the Microlife WatchBPO3 device (20 min intervals during an entire 44 h interdialytic period). The diagnostic thresholds of hypertension were ≥140/90 mmHg for predialysis, ≥130/80 mmHg for postdialysis and ≥130/80 mmHg for 44 h ambulatory BP, respectively. Patients receiving ≥1 antihypertensive medication also were classified as hypertensives. **Results:** The prevalence of hypertension was 88.6% by predialysis, 92.9% by postdialysis and 90.0% by ambulatory BP measurements. In all, 87.1% of patients were being treated for hypertension. When the combination of predialysis and 44 h ambulatory BP was evaluated, the prevalence of sustained normotension, white-coat, masked and sustained hypertension was 52.9%, 21.4%, 5.7% and 20.0%, respectively. A similar distribution of patients into these phenotypes was observed when postdialysis BP was used for the classification of the severity of hypertension (50.0%, 24.3%, 5.7% and 20.0% for sustained normotension, white-coat, masked and sustained hypertension, respectively). Interdialytic ABPM revealed that just one patient had abnormal BP solely during the daytime period. Conversely, isolated nocturnal hypertension was diagnosed in 27.1% of patients. **Conclusions:** This study shows that among patients on hemodialysis, peridialytic BP is an inaccurate proxy of interdialytic ambulatory BP. In approximately 30% of patients, there is discordance between routine peridialytic recordings and interdialytic ABPM for the diagnosis of hypertension. ABPM also facilitates the diagnosis of isolated nocturnal hypertension, which is another frequent BP phenotype in this high-risk patient population.

## 1. Introduction

The epidemiology of hypertension among patients on hemodialysis cannot be objectively described, mainly due to difficulties in the accurate assessment of blood pressure (BP) [1,2]. Most of the currently available evidence is derived from studies that were based on non-standardized (routine) BP recordings taken immediately before or after dialysis [3,4,5]. An inherent methodological limitation of these studies is the fact that predialysis and postdialysis BP measurements are not reliable in diagnosing hypertension and exhibit very high within-subject and between-subject variability. It has to be noted that the diagnostic performance of peridialytic recordings is not substantially improved, even when BP is measured with a standardized methodology [6]. In sharp contrast, studies have shown that relative to peridialytic BP recordings, measurements obtained outside of dialysis are stronger determinants of hypertension-mediated target-organ damage and more powerful predictors of all-cause death risk [7,8,9,10]. On this basis, a more accurate evaluation of the severity of hypertension in this population can be achieved, when the technique of ambulatory BP monitoring (ABPM) is used for the assessment of BP over an entire interdialytic interval [11]. Importantly, prior studies that incorporated the method of interdialytic ABPM for the evaluation of hypertension in hemodialysis patients are very few in number [12,13]. These studies included different thresholds to define abnormal BP and were conducted in patients with considerably variable racial and clinical characteristics [12,13]. It is therefore not surprising that these studies provided different estimates of the proportion of hypertensive patients with adequately controlled ambulatory BP (i.e., 38% versus 28.7% in the first and second study, respectively [12,13].

Accordingly, the primary objective of the present study is to investigate the prevalence and control rates of hypertension in a sample of 70 stable hemodialysis patients using 2-week averaged peridialytic BP recordings and the reference-standard technique of interdialytic ABPM. As secondary objectives, we explore the agreement between peridialytic and ambulatory BP measurements in the diagnosis of hypertension and we provide prevalence estimates for particular BP phenotypes, such as white-coat, masked, isolated daytime and isolated nocturnal hypertension.

## 2. Materials and Methods

### 2.1. Study Population

Our study follows a cross-sectional design. In this analysis, we enrolled patients with kidney failure treated with maintenance thrice-weekly hemodialysis in 2 centers in Northern Greece (AHEPA University Hospital of Thessaloniki and Therepeutiki Dialysis Center). Patients were eligible in our study, if they met the below-mentioned selection criteria: (i) age of 18 years or older; (ii) treatment with in-center hemodialysis 3 times weekly over a period of ≥3 months prior to study enrollment; (iii) willingness to participate in the study, as documented by a signed informed consent. The prespecified exclusion criteria of our study were the following: (i) chronic atrial fibrillation or other chronic cardiac arrhythmia; (ii) functioning or non-functioning arteriovenous fistula or graft in both arms that limits the possibility for accurate assessment of BP; (iii) modification in the hemodialysis regimen or changes in dry-weight and prescribed BP-lowering medications during the last 2 weeks; (iv) history of a recent infectious or bleeding complication; (v) recent hospitalization for acute myocardial infarction, unstable angina, acute decompensated heart failure or stroke; (vi) severe obesity with a body mass index (BMI) ≥ 40 kg/m2.

The protocol of our study was conducted in accordance with the Declaration of Helsinki and its latest Amendments. Signed informed consent was obtained from all study participants before enrollment. Our research protocol was reviewed and received approval by the ethics committee of School of Medicine, Aristotle University of Thessaloniki.

### 2.2. BP Measurements

#### 2.2.1. Interdialytic ABPM

ABPM was carried out with the use of a validated oscillometric arm device (Microlife WatchBPO3, Microlife, Widnau, Switzerland) [14]. BP recordings were initiated immediately after the completion of the second dialysis session of the week and terminated shortly before the next dialysis session. The ABPM device was programmed to obtain BP measurements at 20 min intervals and patients were instructed to remain still with the forearm extended during each measurement [15]. ABPM data were included in the analysis, if ≥ 70% of the scheduled measurements were valid, with ≤2 non-consecutive daytime hours (07:00–23:00) with <2 valid recordings and ≤1 nighttime hours (23:00–07:00) without valid recording [15]. We calculated average values for ambulatory BP recorded over the entire 44 h interdialytic period, as well as separately for BP recordings obtained during the daytime and nighttime periods.

#### 2.2.2. Routine Peridialytic BP Recordings

BP measurements taken by the dialysis-unit staff shortly before and shortly after each dialysis session were collected prospectively. These BP recordings were obtained using automatic oscillometric devices (not necessarily fulfilling international validation criteria) and without a standardized technique (i.e., a prespecified 5 min sitting rest period), according to the everyday clinical practice in each dialysis unit. Routine peridialytic BP recordings were averaged over 2 weeks. Accordingly, for each patient, we used 6 consecutive BP recordings taken before the initiation and 6 after the completion of dialysis for the calculation of average values.

### 2.3. Definitions

**Figure 1:**
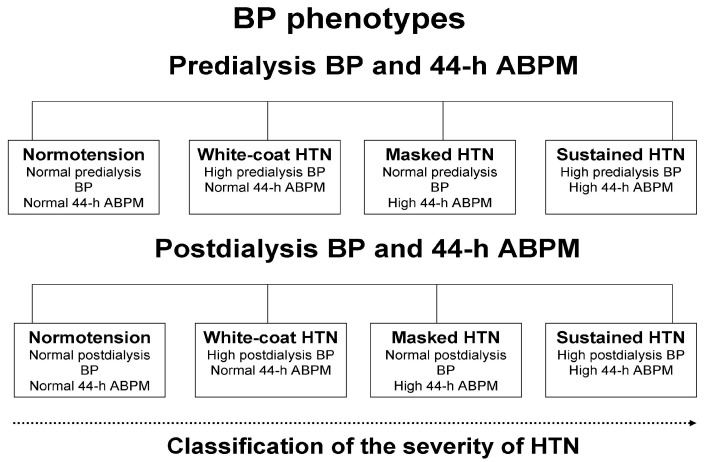
Classification of hypertension into phenotypes with the concomitant evaluation of peridialytic blood pressure recordings and interdialytic ambulatory blood pressure monitoring. **Abbreviations:** ABPM = ambulatory blood pressure monitoring; BP = blood pressure; HTN = hypertension.

The 2023 guidelines of the European Society of Hypertension (ESH) were used for the definitions of ambulatory hypertension in the present analysis [16]. Patients were classified as “hypertensives” in case of: (i) 2-week averaged predialysis BP ≥ 140/90 mmHg or current treatment with at least 1 antihypertensive agent; (ii) 2-week averaged postdialysis BP ≥ 130/80 mmHg or current treatment with any BP-lowering medication; (iii) 44 h interdialytic ambulatory BP ≥ 130/80 mmHg or current use of any antihypertensive medication [16]. The lower BP threshold for postdialysis measurements was chosen as a correction factor for the hemodynamic response of patients to ultrafiltration during the hemodialysis procedure. Hypertensive patients receiving at least 1 antihypertensive medication were classified as “treated hypertensives”. For the purposes of this analysis, loop diuretics were not considered as a separate antihypertensive drug category, given their unproven BP-lowering efficacy in patients with kidney failure. The “control of hypertension” was calculated as the proportion of hypertensive patients achieving (i) 2-week averaged predialysis BP < 140/90 mmHg; and (ii) 2-week averaged postdialysis BP < 130/80 mmHg; (iii) or 44 h interdialytic ambulatory BP < 130/80 mmHg. As shown in Figure 1, for the classification of the severity of hypertension, we defined the following phenotypes, taking into consideration peridialytic BP recordings in combination with 44 h interdialytic ambulatory BP: (i) sustained normotension (predialysis BP < 140/90 mmHg and 44 h BP < 130/80 mmHg or postdialysis BP < 130/80 mmHg and 44 h BP < 130/80 mmHg); (ii) white-coat hypertension (predialysis BP ≥ 140/90 mmHg and 44 h BP < 130/80 mmHg or postdialysis BP ≥ 130/80 mmHg and 44 h BP < 130/80 mmHg); (iii) masked hypertension (predialysis BP < 140/90 mmHg and 44 h BP ≥ 130/80 mmHg or postdialysis BP < 130/80 mmHg and 44 h BP ≥ 130/80 mmHg); (iv) sustained hypertension (predialysis BP ≥ 140/90 mmHg and 44 h BP ≥ 130/80 mmHg or postdialysis BP ≥ 130/80 mmHg and 44 h BP ≥ 130/80 mmHg). The diagnostic thresholds for abnormal BP during daytime and nighttime periods of interdialytic ABPM were ≥ 135/85 mm Hg and ≥ 120/70 mm Hg, respectively [16]. Patients with abnormal BP during nighttime but normal BP during daytime were categorized as having “isolated nocturnal hypertension”. The reverse phenotype of “isolated daytime hypertension” was diagnosed in patients with abnormal BP during daytime but normal BP during nighttime.

### 2.4. Statistical Analysis

The normality of distribution for quantitative variables was assessed with the Kolmogorov–Smirnov test. Quantitative variables are reported as mean ± standard deviation (mean ± SD) or median (range), according to the normality of distribution of each variable. Qualitative variables are reported as frequencies and percentages (n,%). For comparisons between patients with normal and abnormal 44 h interdialytic ambulatory BP, we used the independent Student’s *t*-test or the Mann–Whitney U-test, where appropriate. Receiver operating curve (ROC) analysis was applied to test the accuracy of 2-week averaged predialysis BP at the threshold of 140/90 mmHg and 2-week averaged postdialysis BP at the threshold of 130/80 mmHg in detecting a mean 44 h interdialytic ambulatory BP ≥ 130/80 mmHg [17]. The sensitivity, specificity and positive and negative predictive values of predialysis and postdialysis BP was also assessed. The agreement between peridialytic and interdialytic ambulatory recordings in the identification of patients with abnormal BP levels was explored with the Cohen’s k-coefficient. To explore potential factors associated with inadequate control of interdialytic ambulatory BP, we performed univariate and multivariate binary logistic regression analysis. Variables were tested for interaction and were inserted in the multivariate model if the *p* value in univariate analysis was <0.20. For regression analysis, we report crude and adjusted odds ratios (OR) and the corresponding 95% confidence interval (CI). Probability values of *p* < 0.05 (two-tailed) were considered as statistically significant. The statistical analysis was performed using the Statistical Package for Social Sciences Version 23 (SPSS Inc., Chicago, IL, USA).

## 3. Results

The demographic, clinical and laboratory characteristics of patients enrolled in our study are presented in Table 1. The study population included 70 prevalent hemodialysis patients (45 males, 64.3%) with a mean age of 65.3 ± 13.2 years and a median dialysis vintage of 15 months (range: 3, 117). Our patients had several cardiovascular-related comorbidities, such as dyslipidemia (62.9%), diabetes mellitus (48.6%), coronary artery disease (31.4%) and congestive heart failure (17.1%). The average values of 44 h interdialytic ambulatory BP in the overall study population were 120.6/66.3 mmHg. Of these, 18 patients (25.7%) had an interdialytic ambulatory BP above the diagnostic threshold of hypertension (i.e., ≥ 130/80 mmHg). The vast majority of patients (61/70, 87.1%) were being treated for hypertension with an average number of 1.5 ± 0.6 BP-lowering medications per day. As expected, relative to patients with normal, those with abnormal interdialytic ambulatory BP tended to receive a more intensive antihypertensive drug therapy (1.3 ± 0.5 vs. 1.7 ± 0.8, *p* = 0.125).

**Figure 2:**
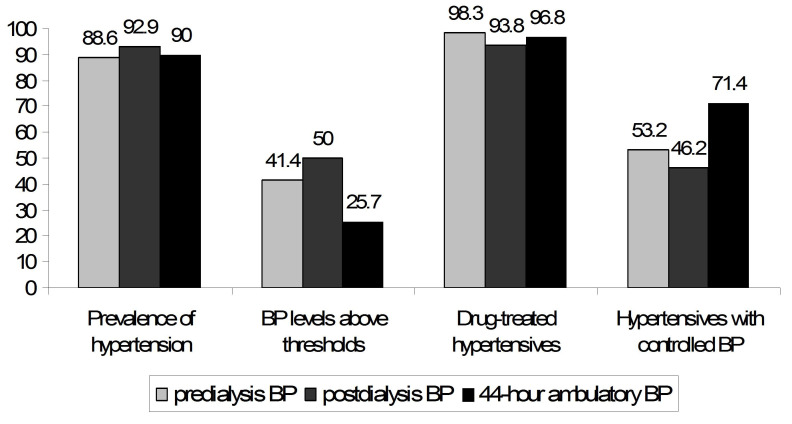
Prevalence and control of hypertension using predialysis BP, postdialysis BP and interdialytic ambulatory BP measurements.

As shown in Figure 2, the prevalence of hypertension in the population under study was high, irrespective of the technique of BP measurement (88.6%, 92.9% and 90.0% by predialysis, postdialysis and interdialytic ambulatory measurements, respectively). Despite the similar rates in the prevalence of hypertension, the proportion of patients with BP levels above the diagnostic threshold of hypertension was higher for predialysis (41.4%) and postdialysis (50.0%) recordings than for interdialytic ambulatory recordings (25.7%). Conversely, the proportion of patients with adequately controlled hypertension was lower when predialysis (53.2%) or postdialysis (46.2%) measurements were used for the assessment of BP than with the use of interdialytic ABPM (71.4%). In multivariate logistic regression analysis, higher hemoglobin levels (adjusted OR: 4902; 95% CI: 1.268–18.953) and the higher number of prescribed antihypertensive medications (OR: 3.835; 95% CI: 1.219–12.068) were the two parameters that were independently associated with inadequate control of interdialytic ambulatory BP (Table 2).

When predialysis and interdialytic ambulatory BP recordings were considered jointly (Table 3), 37 patients (52.9%) were normotensives or had adequately controlled hypertension, 15 patients (21.4%) fulfilled the diagnostic criteria of white-coat hypertension, 4 patients (5.7%) had the reverse phenotype of masked hypertension and the remaining 14 patients (20.0%) had sustained hypertension confirmed by both techniques. When the classification of the severity of hypertension was based on the concomitant evaluation of postdialysis and interdialytic ambulatory BP recordings, a similar distribution of patients into these BP phenotypes was observed (50.0% had sustained normotension or adequately controlled hypertension, 24.3% had white-coat hypertension, 5.7% had masked hypertension and 20.0% had sustained hypertension, respectively).

As shown in Table 4, 16 patients (22.9%) had high BP during the daytime period, and 34 patients (48.6%) had high BP during the nighttime period of interdialytic ABPM, respectively. In all, 15 patients (21.5%) had consistently elevated BP during both daytime and nighttime periods, whereas 35 patients (50.0%) had consistently normal BP during the entire 44 h interdialytic period. The phenotype of isolated nocturnal hypertension was diagnosed in 19 patients (27.1%). Conversely, the phenotype of isolated hypertension during daytime was observed in just 1 out of 70 patients (1.7%).

**Figure 3:**
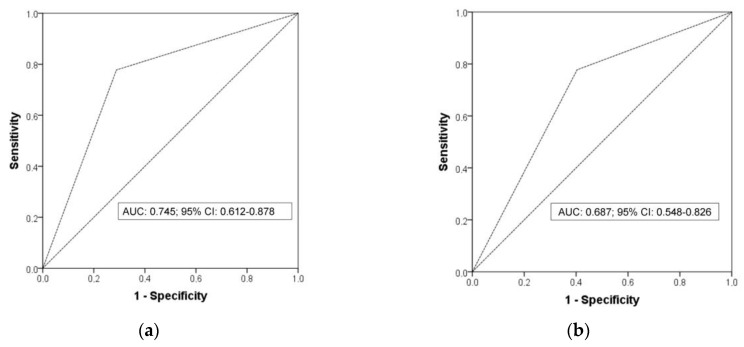
(**a**) The area under the ROC for 2-week averaged predialysis BP at the threshold of 140/90 mmHg in detecting interdialytic ambulatory hypertension. (**b**) The area under the ROC for 2-week averaged postdialysis BP at the threshold of 130/80 mmHg in detecting interdialytic ambulatory hypertension.

With respect to the diagnostic performance of peridialytic BP recordings, the area under the ROC for 2-week averaged predialysis BP at the threshold of 140/90 mmHg in detecting a 44 h interdialytic ambulatory BP ≥ 130/80 mmHg was 0.745 (95% CI: 0.612–0.878) (Figure 3a). The 2-week averaged predialysis BP at this threshold failed to provide a satisfactory combination of high sensitivity (77.8%) and high specificity (71.2%) in the identification of patients with abnormal interdialytic ambulatory BP. The positive and negative predictive values were 48.3% and 90.2%, respectively. The diagnostic agreement between these two methods was poor (k-statistic: 0.408, *p* < 0.001). Similarly, the area under the ROC for 2-week averaged postdialysis BP at the threshold of 130/80 mmHg in diagnosing interdialytic ambulatory hypertension was 0.687 (95% CI: 0.548–0.826) (Figure 3b). The sensitivity, specificity, positive and negative predictive value of 2-week averaged postdialysis BP at this threshold was 77.8%, 59.6%, 40% and 88.6, respectively. Once again, there was very low agreement between postdialysis and interdialytic ambulatory recordings in the detection of patients with BP levels above the diagnostic threshold of hypertension (k-statistic: 0.286, *p* = 0.006).

## 4. Discussion

The present study provides a detailed evaluation of hypertension in a cohort of 70 prevalent hemodialysis patients incorporating two different methods of BP measurement: (i) 2-week averaged peridialytic BP recordings and (ii) 44 h interdialytic ABPM. The main findings of our study are as follows. (i) The prevalence and control rates of hypertension in hemodialysis patients differ depending on the methodology used for the assessment of BP. (ii) In approximately 30% of patients, there is discordance between peridialytic recordings and interdialytic ABPM in the determination of BP control status. (iii) Predialysis and postdialysis BP recordings, even when averaged over six consecutive dialysis sessions, as performed in the present analysis, fail to provide an accurate diagnosis of interdialytic ambulatory hypertension. (iv) The reference-standard technique of ABPM enables the measurement of BP during the period of sleep and facilitates the diagnosis of isolated nocturnal hypertension, which is a frequent BP phenotype in this patient population.

Evidence for the burden of hypertension in hemodialysis patients using the reference-standard method of interdialytic ABPM has been provided by just two prior cross-sectional studies [12,13]. These studies provided variable estimates of the prevalence and control of hypertension. One plausible explanation for this discrepancy is the fact that these studies included patients with variable racial and clinical characteristics. Another possible explanation is the variation between these two studies in the BP monitoring schedule and in the threshold used for the definition of abnormal BP [12,13]. In the first study, 369 hemodialysis patients, predominantly of African American origin, underwent ABPM over an entire 44 h interdialytic period. Hypertension, defined as an average 44 h ambulatory BP ≥ 135/85 mmHg or current use of any BP-lowering medication, was diagnosed in 82% of patients [12]. Although 89% of hypertensives were being treated, the ambulatory BP control rate was 38% [12]. The more intensified antihypertensive drug use in this study appeared to be paradoxically associated with a greater likelihood for inadequately controlled hypertension [12]. In the second study, 389 Caucasian hemodialysis patients underwent 48 h ABPM covering a regular dialysis session and the subsequent interdialytic period [13]. Hypertension, defined as an average 48 h ambulatory BP ≥ 130/80 mmHg or treatment with at least one antihypertensive agent, was prevalent in 84.3% of the study population. Despite the fact that the vast majority of hypertensive patients were receiving antihypertensive drug therapy, only 28.7% of them achieved a 48 h ambulatory BP below the threshold of 130/80 mmHg [13]. The present study confirms that the prevalence of hypertension in hemodialysis patients is very high. However, the proportion of hypertensive hemodialysis patients with adequately controlled interdialytic ambulatory BP in our analysis was higher than that reported in prior ABPM studies. This discrepancy is difficult to be explained. The unexpectedly high rate of controlled ambulatory hypertension may be attributable to the smaller sample size of our study as well as to the therapeutic strategies followed in the two participating dialysis centers (i.e., a more adequate management of hypervolemia).

In the general population, the classification of the severity of hypertension is based on standardized diagnostic criteria [15,16]. However, the classification of hemodialysis patients into different BP phenotypes is controversial, mainly due to the absence of an optimal method to measure and define abnormal BP in the “office” environment. In the present study, we applied two different diagnostic approaches to identify patients with white-coat and masked hypertension. We used 2-week averaged predialysis and 2-week averaged postdialysis BP recordings in combination with 44 h interdialytic ABPM. A similar methodological approach was followed in a prior study incorporating dialysis-unit BP recordings and interdialytic ambulatory BP data obtained from 355 hemodialysis patients [18]. Using a threshold of 140/90 mmHg for predialysis BP and 135/85 mmHg for 44 h interdialytic ambulatory BP, 24% of patients had sustained normotension, 26% had white-coat hypertension, 4% had masked hypertension and 47% had sustained hypertension [18]. When a threshold of 140/80 mmHg for median mid-week dialysis-unit BP was used, the prevalence rates of sustained normotension, white-coat, masked and sustained hypertension was 35%, 15%, 15% and 35%, respectively [18]. In a prospective observational part of this study, an increasing severity of hypertension was associated with a gradual and progressive elevation in the risk of all-cause mortality (multivariate-adjusted hazard ratio: 1.0, 1.3, 1.36 and 1.87 for sustained normotension, white-coat, masked and sustained hypertension, respectively) [18]. Therefore, the phenotypes of white-coat and masked hypertension appear to be prognostically informative in hemodialysis patients, following a trend similar to that seen in the general hypertensive population.

ABPM over an entire interdialytic interval is considered as the “reference-standard” method for the assessment of hypertension in hemodialysis patients for several reasons. As an example, ABPM provides the opportunity to obtain a large number of BP measurements taken during periods of resting and activity, whereas the other techniques provide measurements of BP exclusively during periods of resting [11]. Another unique advantage of ABPM is the fact that this technique enables the measurement of BP during the period of sleep, facilitating in this way the identification of patients with nocturnal hypertension [11]. In the present study, 50% of patients had normal BP during both the daytime and nighttime period and 21.5% of patients had consistently abnormal BP during both periods. Just one patient (1.7%) had abnormal BP solely during the daytime period, whereas the reverse phenotype of isolated nocturnal hypertension was identified in 19 patients (27.1%). The high prevalence of isolated nocturnal hypertension in our cohort should not come as a surprise. The circadian patterns and rhythms of BP in patients with kidney failure are commonly disrupted [19]. The underlying mechanisms include sympathetic overactivity during the night-time period, endothelia dysfunction, insulin resistance, volume overload and sleep-disordered breathing in patients with obstructive sleep apnea [19]. Most importantly, abnormal diurnal variation in BP and nocturnal hypertension are associated with greater progression of target-organ damage as well as with a heightened risk of cardiovascular and all-cause mortality [20,21,22]. Therefore, the diagnosis of these BP phenotypes has important clinical implications for the better prognostication of cardiovascular risk and implementation of therapeutic strategies targeting the normalization of diurnal BP profile. Despite the above-mentioned advantages, interdialytic ABPM still remains a research-grade technique that is not commonly utilized in everyday clinical practice. The potential disadvantages of this method may be the relatively high cost of the equipment, its low availability and the inability to perform repeated ABPM measurements for the assessment and management of hypertension over a long-term period [11].

Important strength of the present study is the concomitant use of peridialytic BP measurements and the reference-standard technique of interdialytic ABPM, a methodology that enabled the accurate classification of patients into different BP phenotypes, such as white-coat, masked and isolated daytime and isolated nocturnal hypertension. However, our analysis has some potential methodological limitations that need to be discussed. First, the sample size of our study is relatively small and patients were enrolled from only two dialysis units in Northern Greece. Accordingly, the reported BP control rates in our study may have been affected by local practices and patterns in the prescription of hemodialysis and our results may not be generalizable to patients with variant racial and clinical characteristics. Larger and multi-center studies are therefore needed for the precise evaluation of the epidemiology of hypertension in the hemodialysis population. Second, predialysis and postdialysis BP recordings were not fully standardized. However, these measurements reflect the daily clinical practice in the assessment of dialysis-unit BP. In addition, we have reasons to believe that the implementation of a standardized protocol for predialysis and postdialysis BP recordings would not substantially modify the results of our study. The diagnostic accuracy of these measurements cannot be strengthened, even when peridialytic BP is recorded with a state-of-the-art technique (i.e., a prespecified 5 min seating rest period before the measurement, the use of validated BP monitors, etch) [23,24]. Third, in the present study, we a priori excluded patients with a recent modification in the prescribed antihypertensive regimen or in the targeted postdialysis weight. This selection criterion facilitates the evaluation of hypertension in stable patients, but may limit the generalizability of our results. Fourth, our study did not include long-term follow-up of the patients and did not explore the prognostic association of several BP phenotypes with the risk for adverse clinical outcomes.

## 5. Conclusions

In conclusion, the present study shows that among patients on hemodialysis, the burden of hypertension is very high, despite the widespread use of antihypertensive drug treatment. The concomitant use of dialysis-unit recordings and interdialytic ABPM enables the detection of white-coat and masked hypertension in approximately 30% of hemodialysis patients. Interdialytic ABPM enables also the diagnosis of isolated nocturnal hypertension, which is another common BP phenotype in hemodialysis patients. Interdialytic ABPM is currently a research technique, but its utilization in daily clinical practice may be helpful for the accurate determination of BP control status and diagnosis of isolated nocturnal hypertension in high-risk hemodialysis patients with evidence of severe target-organ damage. Larger studies incorporating the reference-standard technique of interdialytic ABPM are clearly needed in the future for the precise evaluation of the actual burden of hypertension in this high-risk patient population.

## Figures and Tables

**Table 1:** Clinical characteristics and blood pressure values of study participants.

Clinical Characteristic	Overall	44-Hour Ambulatory BP < 130/80 mmHg	44-Hour Ambulatory BP ≥ 130/80 mmHg	*p* Value
N	70	52	18	-
Age (years)	65.3 ± 13.2	66.7 ± 12.8	61.4 ± 14.1	0.151
Male gender (n,%)	45 (64.3%)	31 (59.6%)	14 (77.8%)	0.254
BMI (kg/m^2^)	25.5 ± 4.1	26.2 ± 4.2	23.3 ± 2.6	0.008
Dialysis vintage (months)	15 (3, 117)	14 (3, 117)	27 (3, 90)	0.407
Comorbidities (n,%)				
Diabetes mellitus	34 (48.6%)	27 (51.9%)	7 (38.9%)	0.417
Coronary artery disease	22 (31.4%)	17 (32.7%)	5 (27.8%)	0.776
Congestive heart failure	12 (17.1%)	11 (21.2%)	1 (5.6%)	0.166
Dyslipidemia	44 (62.9%)	35 (67.3%)	9 (50.0%)	0.259
Basic laboratory parameters				
Hemoglobin (g/dL)	11.6 ± 0.8	11.5 ± 0.8	12.2 ± 0.8	0.008
Serum urea (mg/dL)	135.8 ± 23.8	132.3 ± 23.2	146.2 ± 23.1	0.037
Serum creatinine (mg/dL)	7.3 ± 2.5	7.4 ± 2.4	6.8 ± 2.8	0.420
Serum albumin (g/dL)	4.2 ± 0.4	4.1 ± 0.3	4.2 ± 0.4	0.809
BP values				
44 h ambulatory SBP (mmHg)	120.6 ± 15.2	114.2 ± 10.8	139.1 ± 9.6	<0.001
44 h ambulatory SBP (mmHg)	66.3 ± 10.1	62.6 ± 8.3	77.0 ± 6.5	<0.001
2-week averaged predialysis SBP (mmHg)	134.2 ± 14.2	130.7 ± 12.9	144.3 ± 13.1	<0.001
2-week averaged predialysis DBP (mmHg)	76.6 ± 9.2	74.8 ± 9.0	81.7 ± 7.7	0.005
2-week averaged postdialysis SBP (mmHg)	126.7 ± 15.8	122.9 ± 13.5	137.9 ± 16.9	<0.001
2-week averaged postdialysis DBP (mmHg)	75.8 ± 10.3	74.1 ± 10.4	80.4 ± 8.7	0.016
Antihypertensive drug use (n,%)	61 (87.1%)	45 (86.5%)	16 (88.9%)	0.799
Average number of BP-lowering medications in users (n,%)	1.5 ± 0.6	1.3 ± 0.5	1.7 ± 0.8	0.125

**Abbreviations**: BMI = body mass index; BP = blood pressure; SBP = systolic blood pressure; DBP = diastolic blood pressure.

**Table 2:** Univariate and multivariate logistic regression analysis of factors associated with inadequate control of interdialytic ambulatory BP.

Parameter	Univariate Analysis			Multivariate Analysis		
	Crude OR	95% CI	*p* Value	Adjusted OR	95% CI	*p* Value
Age (per 1 year higher)	0.973	0.934–1.013	0.18	0.954	0.903–1.009	0.10
Gender (male vs. female)	2.333	0.661–8.235	0.19	2.640	0.439–15.888	0.29
BMI (per 1 kg/m^2^ higher)	0.810	0.670–0.979	0.03	0.739	0.581–1.081	0.14
Dialysis vintage (per 1 month higher)	1.002	0.983–1.021	0.84	-	-	-
Current smoker (yes vs. no)	1.132	0.308–4.166	0.85	-	-	-
History of diabetes mellitus (yes vs. no)	1.643	0.540–5.002	0.38	-	-	-
History of coronary artery disease (yes vs. no)	1.174	0.350–3.934	0.79	-	-	-
History of dyslipidemia (yes vs. no)	2.000	0.657–6.084	0.22	-	-	-
History of heart failure (yes vs. no)	3.679	0.425–31.768	0.24	-	-	-
Hemoglobin (per 1 g/dL higher)	3.227	1.334–7.805	0.01	4.902	1.268–18.953	0.02
Serum albumin (per 1 g/dL higher)	1.273	0.257–6.308	0.77	-	-	-
Number of antihypertensives (per 1 agent higher)	2.307	0.955–5.574	0.06	3.835	1.219–12.068	0.02

**Abbreviations:** BMI = body mass index; CI = confidence interval; OR = odds ratio.

**Table 3:** Hypertension phenotypes identified using predialysis, postdialysis and ambulatory blood pressure measurements.

Blood Pressure Phenotype	Predialysis and Ambulatory	Postdialysis and Ambulatory
Normotension or controlled hypertension, n (%)	37 (52.9%)	35 (50.0%)
White-coat hypertension, n (%)	15 (21.4%)	17 (24.3%)
Masked hypertension, n (%)	4 (5.7%)	4 (5.7%)
Sustained hypertension, n (%)	14 (20.0%)	14 (20.0%)

**Table 4:** Patients with daytime and nighttime BP above the thresholds of 135/85 and 120/70 mmHg, respectively.

		Nighttime BP Levels		Total
		≥120/70 mmHg	<120/70 mmHg	
Daytime BP levels	≥135/85 mmHg	15 (21.5%)	1 (1.4%)	16 (22.9%)
	<135/85 mmHg	19 (27.1%)	35 (50.0%)	54 (77.1%)
Total		34 (48.6%)	36 (51.4%)	70 (100%)

## Data Availability

The data used in this study are not publicly available due to privacy and ethical concerns. These data may be shareable after contacting the corresponding author.

## Abbreviations

The following abbreviations are used in the manuscript:

ABPM	Ambulatory blood pressure monitoring
BP	Blood pressure
BMI	Body mass index
CI	Confidence interval
DBP	Diastolic blood pressure
ESH	European Society of Hypertension
ROC	Receiver operator characteristic
SBP	Systolic blood pressure
